# Management of bleeding and invasive procedures in patients treated with anti–factor XI(a) anticoagulants: proposals from the French Working Group on Perioperative Haemostasis and French Society of Thrombosis and Haemostasis

**DOI:** 10.1016/j.rpth.2025.102860

**Published:** 2025-04-17

**Authors:** Anne Godier, Dominique Lasne, Anne–Céline Martin, Gilles Pernod, Pierre Albaladejo, Emmanuel De Maistre, Pierre Fontana, Thomas Lecompte, Grégoire Le Gal, Jerrold H. Levy, Mikael Mazighi, François Mullier, Philippe Nguyen, Stéphanie Roullet, Jean-François Schved, Pierre Sié, Normand Blais, Isabelle Gouin-Thibault, Sophie Susen, French Working Group on Perioperative Haemostasis (Groupe d’Intérêt en Hémostase Périopératoire)

**Affiliations:** 1 Université Paris-Cité, AP-HP, European Hospital Georges Pompidou, Department of Anesthesiology and Critical Care, INSERM U970, Paris Cardiovascular Research Center, Paris, France; 2 Department of Laboratory Haematology, AP-HP, Hôpital Necker-Enfants Malades, Paris, France Institut national de la santé et de la recherche médicale (INSERM) UMRS_1176, Le Kremlin Bicêtre, France; 3 Department of Cardiology, Université Paris-Cité, AP-HP, European Hospital Georges Pompidou, Advanced Heart Failure Unit, Institut national de la santé et de la recherche médicale (INSERM U970), Paris Cardiovascular Research Center, Paris, France; 4 Department of Vascular Medicine, Univ Grenoble-Alpes, University Hospital Grenoble-Alpes, F-CRIN INNOVTE Network, Grenoble, France; 5 Department of Anaesthesia and Critical Care, Grenoble Alpes University Hospital, Grenoble, France; 6 Department of Laboratory Haematology, Hôpital Le Bocage, CHRU de Dijon, Dijon, France; 7 Division of Angiology and Haemostasis, University Hospitals of Geneva, Geneva, Switzerland; 8 Angiology Division, CHRU de Nancy, Lorraine University, Nancy, France; 9 Department of Medicine, University of Ottawa, Ottawa, Ontario, Canada; 10 Departments of Anesthesiology, Critical Care and Surgery, Duke University School of Medicine, Durham, North Carolina, USA; 11 Neurology Department, Université Paris-Cité, Hôpital Lariboisière, Assistance Publique - Hôpitaux de Paris (AP-HP), FHU Neurovasc, Paris, France; 12 Interventional Neuroradiology Department, Hôpital Fondation A. de Rothschild, Paris, France; 13 Institut national de la santé et de la recherche médicale (INSERM) U1144, Institut Universitaire de France, Paris, France; 14 Namur Thrombosis and Hemostasis Center (NTHC), Centre Hospitalier Universitaire (CHU) UCL Namur, Namur Research Institute for Life Sciences (NARILIS), Université catholique de Louvain, Yvoir, Belgium; 15 Institute of Experimental and Clinical Research, UCLouvain, Belgium; 16 Reims Champagne Ardenne University, Reims, France; 17 Department of Anesthesiology and Critical Care, Assistance Publique - Hôpitaux de Paris (AP-HP), Hôpital Paul Brousse, Villejuif, France; 18 Université Paris-Saclay, Institut national de la santé et de la recherche médicale (INSERM), UMRS_1176, Le Kremlin-Bicêtre, France; 19 Montpellier University, Montpellier, France; 20 Toulouse University Hospital, Toulouse, France; 21 Hematology and Oncology Service, Department of Medicine, Centre Hospitalier Universitaire de Montreal, Montreal, Canada; 22 Department of Laboratory Hematology, Pontchaillou University Hospital, IRSET-INSERM-1085, Univ Rennes, Rennes, France; 23 Hematology and Transfusion Department, Lille University Hospital, Institut Pasteur de Lille, Institut national de la santé et de la recherche médicale (INSERM), U1011-European Genomic Institute for Diabetes (EGID), Université de Lille, Lille, France

**Keywords:** bleeding, guidance, invasives procedures, anticoagulation reversal

## Abstract

**Background:**

Several anti-FXI(a) agents with distinct mechanisms of action and pharmacological properties are currently under clinical development. While these anticoagulants are not yet available, there is a need to address bleeding risk management for patients already enrolled in phase III trials. These patients may face elective or unplanned invasive procedures and bleeding events in anticipation of marketing authorization.Experience from managing patients with inherited FXI deficiency, along with data from early clinical trials, suggests that the bleeding risk associated with anti-FXI(a) is likely to be low but can vary depending on the clinical situation. Anti-FXI(a) reversal options include tranexamic acid, FXI concentrates, and recombinant activated factor VII. However, these options may not always be suitable, can be expensive, and may carry a thrombotic risk.

**Objectives:**

The French Working Group on Perioperative Haemostasis (Groupe d’Intérêt en Hémostase Péri-opératoire (GIHP)) and the French Society of Thrombosis and Haemostasis (SFTH) aimed to develop proposals to manage bleeding and invasive procedures in patients treated with anticoagulants targeting Factor XI or XIa (anti-FXI(a)).

**Methods:**

Literature review and development of practical guidelines by an expert panel.

**Results:**

We propose pragmatic recommendations for optimizing safety in patients treated with anti-FXI(a), considering bleeding and thrombosis risks, the drug’s mechanism of action, and available reversal options.

**Conclusion:**

These proposals will be re-evaluated as more data becomes available. The implementation of a registry for managing anti-FXI(a) anticoagulants in patients undergoing invasive procedures or experiencing bleeding complications is needed.

## Essentials

• Managing bleeding risk in patients on anti-FXI(a) lacks clear practical protocols.
• Experience suggests anti-FXI(a) has a low bleeding risk, but its reversal options raise questions.
• As part of risk management, experts propose guidelines using trial data and FXI deficiency.
• The above options help clinicians in unstudied cases and highlight research gaps.

## 1 Introduction

Anticoagulants play a key role in the prevention and treatment of arterial and venous thromboembolism events. However, in their main indication (ie, stroke prevention in atrial fibrillation [AF] or venous thromboembolism [VTE]), the inherent bleeding risk associated with all anticoagulants, including direct oral anticoagulants (DOACs), limits their prescription in a substantial proportion of eligible patients.

Inhibiting factor (F)XI pathway is a promising option as it is important for thrombosis but plays a minor role in hemostasis. Therefore, anticoagulants targeting FXI or XIa (anti-FXI[a]) have been developed with the aim of having drugs with antithrombotic activity at least as effective as DOACs anti-IIa or anti-Xa agents, but with a significantly lower risk of bleeding.

FXI plays a role in physiological hemostasis by strengthening the clot. After vascular injury, the tissue FVIIa complex initiates the activation of the coagulation protease cascade, leading to the burst of thrombin generation and fibrin formation. The amplification feedback loop, particularly via FXI activated by thrombin, strengthens and consolidates clot formation, contributing to clot formation. In addition, the high concentration of thrombin is thought to play a role in protecting clots from premature lysis through activation of thrombin-activable fibrinolysis inhibitor [[1], [2], [3]].

In contrast, in thrombosis, FXI activation is critical for clot propagation beyond the site of injury, ultimately leading to thrombus formation. The contact phase can be activated upon contact with damaged endothelium, atherosclerotic plaque disruption, activated cells or extracellular vesicles, and artificial surfaces. FXI links the contact phase to intrinsic tenase, being activated by FXIIa and in turn activating FIX to FIXa [2]. FXI can also be activated by thrombin via the positive feedback loops of the coagulation system, thus contributing to thrombus formation.

FXI also binds specifically and reversibly to high affinity sites (Kd: 10^−8^ M) on the surface of stimulated platelets [4], which enhances FXI activation by thrombin. Activated platelets secrete polyphosphates, enhancing by 3000-fold thrombin activation of FXI and making this back-activation reaction a physiologically relevant contributor to sustained thrombin generation [5].

In addition, a platelet FXI has been reported as an alternatively spliced product of FXI gene specifically expressed within megakaryocytes and platelets. Of interest, functionally active platelet FXI is differentially expressed on platelet membranes constitutively and in a concentration-dependent manner after activation in the absence of detectable plasma FXI [6]. This may contribute to the poor correlation of plasma FXI level with bleeding outcomes in hereditary FXI deficiency. Indeed, inherited FXI deficiency, even in the case of major deficiency, is characterized by a mild to moderate bleeding tendency, mainly in regions where the fibrinolytic system is physiologically very active (mouth, upper airways, and urinary tract). In contrast, the thrombotic risk is reduced especially in cases of venous thromboembolism and stroke [7].

Several strategies have been proposed to inhibit FXI and/or FXIa, of which 3 are currently in advanced clinical development: antisense oligonucleotides (ASOs), monoclonal antibodies (mABs), and small molecules (Table). Importantly, these therapeutic classes differ in their mechanisms of action and pharmacologic properties. Published phase II and phase III data have confirmed a low risk of bleeding, but their antithrombotic efficacy in AF is still controversial, particularly for small-molecule anticoagulant asundexian [9]. Nevertheless, it is likely (at the time of writing) that these anticoagulants, or at least some of them, will reach the market in the next few months or years.

**Table:** Main characteristics of anti-FXI(a) anticoagulants [3,8].

Characteristics	Antisense oligonucleotide	Monoclonal antibodies (IgG)			Small molecules	
	Fesomersen (IONIS-FXI-LRx)	Abelacimab	Osocimab	Xisomab (gruticibart)	Asundexian	Milvexian
Mechanism of action	Reduces FXI synthesis by the liver • FXI deficiency	Binds to the catalytic domain of FXI and FXIa	Binds to the catalytic domain of FXIa	Binds to the apple 2 domain of FXI(a)	Inhibits FXIa catalytic active site	Inhibits FXIa catalytic active site
Indications under development	ESKD, TKR	AF, CAT, TKR	ESKD, TKR	CAT, ESKD, TKR	AF^a^, stroke, ACS	AF, stroke, AMI, TKR
Administration route and frequency	Subcutaneous weekly to monthly	Subcutaneous or IV	IV	IV	Oral, once daily	Oral, twice daily
Half-life	Effect may persist for weeks to months after discontinuation	25-30 d	30-44 d	121 h (for 1 dose of 5 mg/kg)	14.2-17.4 h	11.4-18.1 h
Hepatic metabolism	No	No	No	No	CYP3A4/3A5	CYP3A4/3A5
Renal excretion	No	No	No	No	<15%	<20%

ACS, acute coronary syndrome; AF, atrial fibrillation; AMI, acute myocardial infarction; CAT, cancer-associated thrombosis; ESKD, end-stage kidney disease; IV, intra venous; TKR, total knee replacement; VTE, venous thromboembolism.

a Study stopped early due to lack of efficacy.

A significant proportion of patients treated with anti-FXI(a) anticoagulants will inevitably be exposed to various critical situations, spontaneous or traumatic bleeding, elective or unplanned surgery, or invasive procedures. Unfortunately, data from patients enrolled in trials evaluating anti-FXI(a) anticoagulants and exposed to invasive procedures are not yet fully available. Periprocedural bleeding among patients undergoing invasive procedures included in Abelacimab Compared with Open-Label Rivaroxaban in Patients with Atrial Fibrillation–Thrombolysis in Myocardial Infarction 71 trial evaluating abelacimab in AF has recently been presented [8]. Approximately 35% of patients underwent invasive procedures during the study, with 1 in 4 being unplanned. While the overall rate of procedural bleeding was low, more than 3 quarters of the procedures were low risk and were procedures that are often performed without stopping anticoagulation (eg, coronary angiography). However, among the few patients undergoing high-risk procedures, those treated with abelacimab experienced an unexpected higher percentage of bleeding events than patients treated with rivaroxaban (4.7% vs 0).

Initial experience with unplanned surgery and bleeding complications in patients enrolled in phase III trials, despite being managed in expert centers, shows that trial protocols are unclear [[9], [10], [11]]. This limited experience also highlights significant gaps in our understanding of the optimal approaches to managing such interventions particularly regarding bleeding risk assessment, laboratory testing, and modalities to improve hemostasis or reverse anti-FXI(a) effects [8].

Although some expert groups have addressed the issue of management attitudes, many aspects remain to be considered to establish appropriate, affordable, and practical approaches to patient care [7,12,13]. In addition, this is a new class of antithrombotic agents with molecules that have very different mechanisms of action and pharmacologic properties, and it is critical to provide pragmatic management protocols that need to be re-evaluated.

As part of a “risk management” process and in anticipation of marketing authorization, consultations were held within the Working Group on Perioperative Haemostasis (Groupe d’intérêt en Hémostase Périopératoire) and the group TITANs (Thrombosis, antIcoagulanTs and ANtiplatelet agents) from the French Society of Thrombosis and Haemostasis regarding the management of bleeding risk of these patients. Regarding the method, a first version based on the recommendations for patients with inherited FXI deficiency [14] was adapted for anti-FXI(a) anticoagulants using pharmacologic studies and the phase I and II trials. The text was then submitted to the members of the Groupe d’intérêt en Hémostase Périopératoire and the French Society of Thrombosis and Haemostasis for several rounds of critical analysis until a consensus was reached, which is presented below.

## 2 Laboratory Testing

All anti-XI(a) anticoagulants prolong the activated partial thromboplastin time (aPTT) [15]. However, sensitivity of aPTTs (reagents—coagulometers) to FXI deficiencies is highly variable due to different compositions in contact activator and phospholipids [16]. The same would likely be the case with any anti-FXI(a) anticoagulant, as already shown with milvexian [17]. In addition, aPTT has many limitations regarding its clinical relevance to define the bleeding risk.

Measurement of FXI:C (clotting assay), with a turnaround time of 1 to few hours depending on the laboratory, could be clinically useful to document low plasma levels due to decreased synthesis (ASOs). It is useless with the other anti-FXI(a) anticoagulants (mABs, small molecules), which are not expected to induce a decrease in plasma FXI levels but may interfere with FXI and other coagulation factors’ measurement. It is therefore advisable to measure coagulation factors using further dilutions of the patient plasma to overcome any inhibitory effect and avoid misinterpretation [18].

Thrombin generation has been assessed *in vitro* in some studies during the development of anti-FXI(a) anticoagulants under very specific conditions [[19], [20], [21]] but cannot be used in clinical practice.

Regarding point-of-care, activated clotting time (ACT) or viscoelastometric tests, there are too few data to draw conclusions about the effect of anti-FXI(a) anticoagulants.

Although more data are urgently needed to better determine the need for assays, the currently available laboratory tests may still be helpful in managing perioperative or bleeding management in patients receiving anti-FXI(a) anticoagulants as follows:

• A normal aPTT would indicate low or no defect of FXI, providing that the sensitivity of the local aPTT is known.
• For patients treated with ASOs, we propose measuring FXI plasma levels although it is not available everywhere around the clock. It should be used in the same way and with the same limitations as for inherited FXI deficiency. For other anti-FXI(a) anticoagulants, measurement of FXI levels appears to be of little use and may even be misleading due to interferences in the assay [22].
• Tests performed in daily practice that are not affected by anti-FXI(a) anticoagulants include prothrombin time-international normalized ratio, fibrinogen Clauss, and D-dimer.

## 3 Management of the Bleeding Risk: From Inherited FXI Deficiency to anti-FXI(A) Anticoagulants

### 3.1 Management of the bleeding risk in inherited FXI deficiency

The management strategy for the bleeding risk associated with anti-FXI(a) anticoagulants is extrapolated from patients with moderate or severe inherited FXI deficiency who have undergone surgery or experienced bleeding. Although there is significant clinical heterogeneity, patients with FXI deficiency rarely experience spontaneous bleeding but have an increased risk of perioperative bleeding. However, the bleeding tendency is generally mild or moderate, less pronounced than in patients with hemophilia, and varies according to the surgical site. It is lower in bone surgery and higher in sites with high fibrinolytic activity, such as ENT (ear, nose, and throat) and genitourinary tract surgery. As previously discussed, isolated analysis of residual FXI levels, or aPTT, or genotype does not allow estimation of the risk of bleeding.

Surgery performed without prophylaxis in patients with inherited FXI deficiency is associated with a high incidence of bleeding complications [2,23]. Therefore, the pharmacologic management of the bleeding risk relies primarily on tranexamic acid (TXA), adapted in case of renal insufficiency, human recombinant activated FVII (rFVIIa), FXI plasma concentrates (Hemoleven), and/or fresh frozen plasma (FFP) [14]. TXA is widely used because inherited FXI deficiency is associated with increased sensitivity to fibrinolysis. As the increased bleeding risk associated with inherited FXI deficiency is mild to moderate, it can often be controlled with the administration of TXA only [24]. Recommended doses are 15 to 20 mg/kg or 1 g 4 times daily for 5 to 7 days [25]. For surgery with a high risk of bleeding, rFVIIa and FXI plasma concentrates (Hemoleven) are the 2 options. FFP (15-20 mL/kg) may be an alternative if FXI concentrate is not available. The choice between them depends not only on the patient’s medical condition but also on personal experience and the availability of FXI concentrates in the specific country and center. The administration of low doses of rFVIIa (10 to 15 μg/kg as a single injection) in combination with TXA seems to be the most common practice due to product availability and the absence of reported thrombotic risk in treated patients so far [26]. This allows bypassing FXI deficiency and increasing resistance to fibrinolysis as the thrombin generated would enhance thrombin-activable fibrinolysis inhibitor. Conversely, although FXI supplementation seems logical to compensate FXI deficiency, the administration of FXI concentrates has been associated with thrombotic events, leading to specific recommendations: monitoring Hemoleven treatment by a hemostasis specialist, calculating the dosage to achieve a target level of 30% to 40% (0.3-0.4 IU/mL, as 1% = 0.01 IU/mL [1 U/dL]) of FXI in patients with severe deficiency (baseline FXI level < 20%), with a general recovery rate of 2%, an initial recommended dose of 10 to 15 IU/kg with a maximum of 15 IU/kg requiring reassessment, and no reinjection within 24 hours [27].

Other therapeutic proposals have been reported, such as the use of FFP [25], because FXI concentrates are not available in all countries. However, such an approach requires large volumes to be transfused. Apart from a few case reports, there are no data on the use of desmopressin, activated prothrombin complex concentrate (APCC), or PCC, with the latter 2 being proposed as alternatives to bypass FXI(a) inhibition when rFVIIa and FXI are not available. In this case, PCC should be preferred.

### 3.2 Proposals for the management of patients treated with anti-FXI(a) anticoagulants

**Figure 1:**
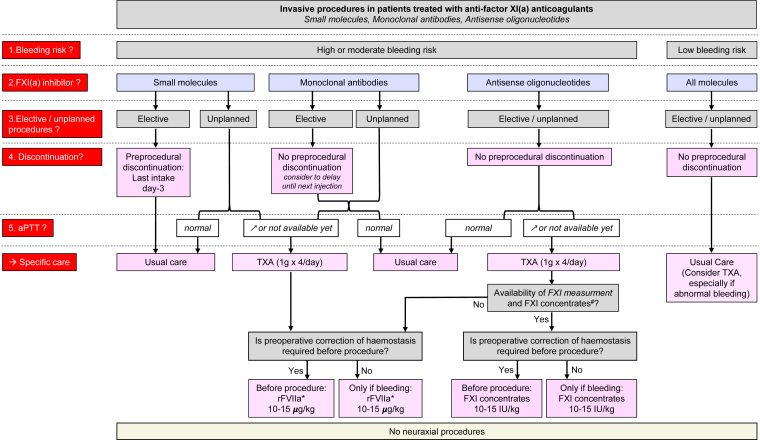
Invasive procedures in patients treated with anti–factor (F)XI(a) anticoagulants. ∗If not available, consider prothrombin complex concentrate (PCC), activated prothrombin complex concentrate (APCC), and fresh frozen plasma (FFP). ^#^Fresh frozen plasma (15-20 mL/kg) may be an alternative to FXI concentrate. aPTT, activated partial thromboplastin time; rFVIIa, recombinant activated FVII; TXA, tranexamic acid.

**Figure 2:**
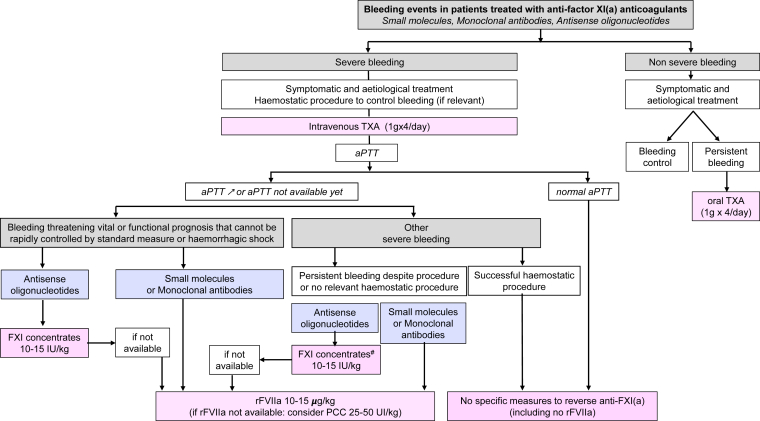
Bleeding management in patients treated with anti–factor (F)XI(a) anticoagulants. ^#^Fresh frozen plasma (15-20 mL/kg) may be an alternative to FXI concentrate. aPTT, activated partial thromboplastin time; PCC, prothrombin complex concentrate; rFVIIa, recombinant activated FVII; TXA, tranexamic acid.

Translating data from inherited FXI deficiency into the context of anti-FXI(a) anticoagulants requires caution and consideration of the mechanism of action of the anticoagulant. The following key points will help define management. The risk of bleeding with anti-FXI(a) anticoagulant is probably lower than that with other anticoagulants. Preoperative discontinuation of treatment only makes sense for molecules with short half-lives (ie, small molecules). As a result, the majority of elective and unplanned procedures are performed on anticoagulation, which is a specific feature of these treatments (ie, ASOs or mABs). There is currently no specific antidote, but the development of one is at an early stage [28]. FXI plasma concentrates can only be considered for patients treated with an ASO, as this situation most closely mimics severe/complete FXI deficiency. They will be ineffective with molecules that target FXI(a) or those that inhibit FXI in a saturating manner. TXA or possibly rFVIIa, which do not specifically target FXI, may be effective with all molecules. The same dosing regimens are proposed as for inherited FXI deficiency. Preliminary data from the Abelacimab Compared with Open-Label Rivaroxaban in Patients with Atrial Fibrillation–Thrombolysis in Myocardial Infarction 71 trial [8] have led us to propose the 2 algorithms shown in Figure 1, Figure 2.

#### 3.2.1 Invasive procedures in patients treated with anti-FXI(a) anticoagulants

For invasive procedures with a low risk of bleeding (dental extraction, carpal tunnel release, gastric or colonic biopsy, etc.), because anti-FXI(a) anticoagulants increase sensitivity to fibrinolysis, a short course of TXA is proposed, either prophylactically or in the event of abnormal bleeding, depending on the procedure and its assessed bleeding risk (Figure 1).

For invasive procedures with a high risk of bleeding, the experience with inherited FXI deficiency suggests that bleeding complications should be prevented [14,25]. Methods to improve hemostasis depend on the specific anti-FXI(a) anticoagulant and its characteristics (Table).

Anticoagulant discontinuation before elective procedures is only possible for small molecules such as milvexian or asundexian, which have half-lives of 11 to 18 hours and 16 to 18 hours, respectively. Pharmacologic data showed that 48 h after the last intake, the concentrations dropped to less than 10% of the *C*_max_ at doses of 25 mg to 100 mg of asundexian [29] and of 20 mg to 200 mg of milvexian [30,31]. Therefore, discontinuation on day 3 before surgery might be proposed. Considering the long half-life of the mABs or ASOs and their anticoagulant effect lasting for several weeks, their discontinuation is not an option so most surgeries would be performed in an anticoagulated patient. In this case, we proposed the use of TXA (Figure 1). In the specific case of mABs, if the procedure could be delayed until just before the next injection, the risk of bleeding would be partially reduced.

If the increased bleeding risk associated with the anti-FXI(a) agent is unacceptable, reversal of its effect should be considered and balanced against the thrombotic risk of the reversal agent:

• In the case of mABs, rFVIIa should be proposed.
• Treatment with ASOs induces a highly variable reduction in FXI levels, which may be less than 0.1 IU/mL (10%) [32]. In this case, FXI concentrates are proposed following the same protocols as for FXI deficiency, as these anticoagulants significantly reduce FXI levels. The thrombotic risk associated with FXI concentrates should be considered, although unknown in these specific populations at cardiovascular risk. Pre- and postadministration FXI monitoring is critical for procedures requiring optimal hemostasis for several days (eg, neurosurgery and complex urological surgery) to guide the rare cases where reinjection may be required after the first day (with a half-life of 48 hours, reinjection should not occur within 24 hours). rFVIIa administration is the alternative to FXI concentrates, as described above.

For unplanned invasive procedures, management depends on the anti-FXI(a) anticoagulant on board, the procedure-associated bleeding risk, and the aPTT. For patients treated with inhibitory small molecules and mABs, rFVIIa and TXA may be proposed to improve hemostasis, whereas there is no place for FXI concentrates. For patients treated with ASOs, FXI concentrates or rFVIIa may be proposed, which are associated with TXA.

Both the perioperative bleeding risk associated with anti-FXI(a) anticoagulants and the safety of rFVIIa and FXI concentrates are insufficiently documented for both elective or unplanned procedures. Therefore, it is proposed not to systematically administer rFVIIa or FXI concentrates preoperatively, but rather to consider the type of procedure; that is, in situations where preoperative correction of hemostasis is required because the procedure involves tissues or organs where bleeding would have serious functional consequences, or where bleeding would be uncontrollable by surgical means (eg, neurosurgery or complex surgery). In such cases, it is proposed that rFVIIa or FXI concentrates be administered preoperatively. Conversely, in situations where bleeding can be monitored and controlled intraoperatively, it is proposed that administration of rFVIIa or FXI concentrates be limited to cases of overt abnormal bleeding.

Although the dose-response curve for aPTT prolongation needs to be assessed for each molecule, a normal aPTT indicates that high doses of anti-FXI(a) anticoagulants can be excluded, regardless of the specific molecule, and leads to usual care.

#### 3.2.2 Bleeding management in patients treated with anti-FXI(a) anticoagulants

Management depends on bleeding severity. Nonsevere bleeding is treated symptomatically and etiologically without correction of hemostasis [33]. In cases of persistent bleeding, the administration of TXAis suggested (Figure 2).

On the opposite, in severe bleeding, symptomatic and etiological treatments are systematically combined with TXA. The duration of TXA treatment is not established, but could be several days, especially when used alone and for patients treated with ASOs.

aPTT measurement guides management: a normal aPTT is unlikely to be associated with a high level of anticoagulation and leads to standard management.

Conversely, prolonged aPTT may indicate the presence of the anticoagulant but does not provide information on the concentration of the drug and the level of anticoagulation. Moreover, a prolonged aPTT may also result from other causes than the presence of anti-XI(a) (bleeding-induced coagulopathy, lupus anticoagulant, etc.).

Regarding reversal, the same options as for invasive procedures are suggested:

• For treatment with small molecules and mABs, rFVIIa is proposed.
• For ASOs, administering low doses of FXI concentrates leads to a partial reversal of anticoagulant effect but may be associated with a thrombotic risk, especially in patients with cardiovascular risk factors. Laboratory measurement of plasma FXI levels may be considered after administration of concentrates, especially in cases of persistent or recurrent bleeding. rFVIIa is the alternative to FXI concentrates.

As a result, the timing of reversal varies according to the severity of bleeding: for bleeding events that threaten vital or functional prognosis and cannot be rapidly controlled with standard measures, or for hemorrhagic shock, urgent treatment is needed, whereas for other severe bleeding events, reversal should be considered if bleeding continues despite hemostatic intervention or if no hemostatic intervention is possible to control the bleeding.

The issue of pharmacologic venous thromboprophylaxis after invasive procedures or bleeding is unsettled. Low-molecular-weight heparin could be considered, particularly if small-molecule treatments such as milvexian or asundexian are discontinued and rFVIIa or FXI concentrates are administered.

#### 3.2.3 Resumption of anti-FXI(a) treatment. Resumption of anti-FXI(a) treatment after surgery or severe bleeding is poorly documented

In the case of small molecules, therapeutic strategies for AF or VTE would be same as for DOACs. After high bleeding risk procedures, therapeutic anticoagulation is usually resumed within 48 to 72 hours. After severe bleeding, the same intervals are usually proposed once the bleeding is controlled. After intracerebral hemorrhage (ICH), the question is unsolved. For VTE, low-molecular-weight heparin could be considered for venous thromboprophylaxis until anti-FXI(a) treatment is resumed.

With long-acting anti-FXI(a) treatment (ie, oligonucleotides or mABs), patients remain anticoagulated by the drug regardless of the management.

## 4 Conclusion

The aforementioned options are only suggestions supported by a low level of evidence. They are intended to help clinicians who are faced with these molecules in situations that have not been rigorously evaluated. They also aim to identify “research gaps to fill” in management of these patients. These suggestions will be re-evaluated as more data are published, and the implementation of registry for the management of anti-FXI(a) anticoagulants in cases of invasive procedures or bleeding complications is needed.
